# Preoperative Patient Guidance and Education in Aesthetic Breast Plastic Surgery: A Novel Proposed Application of Artificial Intelligence Large Language Models

**DOI:** 10.1093/asjof/ojae062

**Published:** 2024-08-13

**Authors:** Jad Abi-Rafeh, Brian Bassiri-Tehrani, Roy Kazan, Heather Furnas, Dennis Hammond, William P Adams, Foad Nahai

## Abstract

**Background:**

At a time when Internet and social media use is omnipresent among patients in their self-directed research about their medical or surgical needs, artificial intelligence (AI) large language models (LLMs) are on track to represent hallmark resources in this context.

**Objectives:**

The authors aim to explore and assess the performance of a novel AI LLM in answering questions posed by simulated patients interested in aesthetic breast plastic surgery procedures.

**Methods:**

A publicly available AI LLM was queried using simulated interactions from the perspective of patients interested in breast augmentation, mastopexy, and breast reduction. Questions posed were standardized and categorized under aesthetic needs inquiries and awareness of appropriate procedures; patient candidacy and indications; procedure safety and risks; procedure information, steps, and techniques; patient assessment; preparation for surgery; postprocedure instructions and recovery; and procedure cost and surgeon recommendations. Using standardized Likert scales ranging from 1 to 10, 4 expert breast plastic surgeons evaluated responses provided by AI. A postparticipation survey assessed expert evaluators' experience with LLM technology, perceived utility, and limitations.

**Results:**

The overall performance across all question categories, assessment criteria, and procedures examined was 7.3/10 ± 0.5. Overall accuracy of information shared was scored at 7.1/10 ± 0.5; comprehensiveness at 7.0/10 ± 0.6; objectivity at 7.5/10 ± 0.4; safety at 7.5/10 ± 0.4; communication clarity at 7.3/10 ± 0.2; and acknowledgment of limitations at 7.7/10 ± 0.2. With regards to performance on procedures examined, the model's overall score was 7.0/10 ± 0.8 for breast augmentation; 7.6/10 ± 0.5 for mastopexy; and 7.4/10 ± 0.5 for breast reduction. The score on breast implant–specific knowledge was 6.7/10 ± 0.6.

**Conclusions:**

Albeit not without limitations, AI LLMs represent promising resources for patient guidance and patient education. The technology's machine learning capabilities may explain its improved performance efficiency.

**Level of Evidence: 4:**

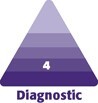

Plastic surgery has a long history of pushing the frontier of medical and surgical innovation.^1^ As of late, the utility of artificial intelligence (AI), a rapidly evolving technology in the field of computer science, has grown in great measure.^2-4^ As plastic surgeons, it remains crucial for us to explore and harness the utility of this evolving technology, while shedding light on its potential detriments to our patients and the specialty at large.^5,6^

AI refers to computer systems that emulate human intelligence and cognitive processes, allowing autonomous perception, knowledge synthesis, and inference of information.^2,7,8^ This was first postulated by Alan Turing in his 1950 seminal work “Computing Machinery and Intelligence.”^7-12^ ChatGPT is an AI-powered chatbot developed by OpenAI (San Francisco, CA) that has taken the world by storm.^13-17^ Several publications have demonstrated its potential to complete an array of cognitively demanding human tasks, such as contributing to scientific manuscripts^13,18,19^ and writing medical board examinations.^20^ As a large language model (LLM), it has natural language processing capabilities that allow it to solicit, interpret, and synthesize human text.^21^ The potential applications of LLMs in plastic surgery are vast^5^; their potential role in patient guidance and patient education is of particular interest to our group. In the present study, the authors explore and objectively evaluate the performance of ChatGPT in answering standardized, simulated questions from the perspective of patients interested in aesthetic breast plastic surgery.

## METHODS

The freely available and patient-accessible version of ChatGPT (ChatGPT-3.5) was queried using simulated interactions from the perspective of patients interested in aesthetic breast plastic surgery, including breast augmentation, mastopexy, and breast reduction. In order to assess the bot's ability to provide patient-specific answers and recommendations, 3 hypothetical and unique patient profiles, with different modifiable and nonmodifiable medical and lifestyle risk factors, were generated and provided for each of the query iterations.

Standardized questions posed to the AI model spanned 7 major categories deemed critical in the preoperative guidance and evaluation process. (Table 1, Appendices A-C) All standardized simulated interactions were performed by a single author (J.A.-R.) during May 2023; the same computer (MacBook Pro, Apple, Inc., Cupertino, CA), browser (Google Chrome, Google Inc., Mountain View, CA), and Internet connection were used. No “alternate answers” were requested at any time to avoid bias. All chats were closed, browser history (including cookies) cleared, and computer restarted before each new session to eliminate any potential impact of locally stored data on the bot's efficiency and performance. The latter included the time required to provide users with responses, and its ability to anticipate questions and preemptively provide answers based on its history of interactions with users.

**Table 1.** Outline of Standardized Questions Posed to ChatGPT From the Perspective of Patients Interested in Breast Augmentation, Mastopexy, and Breast Reduction

Questions	Answering capabilities
*Aesthetic needs inquiry: awareness of available procedures*	
I have some questions about breast plastic surgery I’d like to ask you. Feel free to ask me more questions if you need, so you can give me the best answers. I’m bothered by… (patient-specific aesthetic concerns). What are my options?	Able to capture and synthesize patients' aesthetic concerns, present, and contrast different surgical options and potential procedure combinations (eg, augmentation with implants + fat transfer, or augmentation mastopexy) Emphasizes the need to consult with a board-certified plastic surgeon to assess individual patient needs and goals, obtain a medical history, perform a physical examination, and discuss potential risks, recovery, and expected results
*Patient candidacy and procedure indications*	
I am a (__)-year-old female, with (medical and lifestyle risk factors). Am I a good candidate for this surgery?	In response to simulated patient profiles with different lifestyle and medical risk factors, ChatGPT is generally capable of counseling patients on elements that may put them at greater risk for surgery and/or affect their candidacy for surgical procedures However, its ability to identify specific “red flag” risk factors, of greater relevance than others for specific procedures (eg, cocaine use and breast reductions), may be limited Encourages patients to disclose all shared information with their plastic surgeon and generally defers to plastic surgeons for assessment on whether or not patients are good candidates for surgery
*Procedure safety and risks*	
What are the risks associated with (procedure)? Can you give me specific estimates, and an overall complication rate? Are there long-term complications I should know about? Can I have kids and breastfeed after (procedure)?” Will (procedure) increase my chances of getting or detecting breast cancer in the future? Is it safe to combine (procedure) with other procedures?	Capable of providing a comprehensive yet incomplete list of possible complications associated with different procedures of interest Inconsistent in its ability to provide specific risk estimates, despite being prompted; capable of sharing incidences associated with breast augmentation complications, but not complications associated with mastopexy or breast reduction Capable of discussing potential implications of certain complications (eg, implant loss with severe infections or reoperations due to asymmetry) Encourages patients to discuss risks further with their plastic surgeon to take into account their medical history, lifestyle risk factors, and individual circumstances that may have an impact on incidence Capable of answering general questions regarding risks on lactation and breast cancer screening; encouraging good practices such as self-breast examinations and follow-up with primary care providers
*Procedure information, steps, and techniques*	
Can you explain to me the steps of how (procedure) is performed? Are there different techniques of how (procedure) can be performed? Where will my scars be?	Capable of providing patients with a general structured outline of surgical steps within a simplified, patient-appropriate level Elements discussed include anesthesia and its possibilities (general vs sedation), incision placement options, breast implant pocket considerations, breast implant options, tissue removal, reshaping, and nipple repositioning in mastopexy/breast reduction, as well as incision closure and dressings A limited discussion of different techniques within each procedure is provided (eg, lollipop vs vertical vs circumareolar lifts)
*Implant-specific knowledge*	
You mentioned different kinds of implants, places of incision placement, and pockets where the implants will go. Can I choose what I want? Can you tell me the advantages and disadvantages of each? Are implants considered to be safe? Do implants cause any diseases or cancer? What's the difference between textured and smooth implants? Will I need my implants changed in the future?	Capable of providing a thorough yet incomplete comparative primer on differences between saline and silicone implants, with relative advantages and disadvantages of each Also capable of contrasting subglandular vs subpectoral pocket placement choices, as well as inframammary, periareolar, and transaxillary incisions, with relative advantages and disadvantages of each, and good yet imperfect accuracy and comprehensiveness Capable of reassuring patients on the safety of breast implants with reference to approval by regulatory authorities (FDA), and discusses risk of BIA-ALCL, but with no mention of other possible capsular malignancies, or BII. Accurate information on implant longevity, and importance of regular follow-up with plastic surgeons Compares textured vs smooth implants with relevance to their relative characteristics, and with good comprehensiveness, but omits key information on BIA-ALCL in this discussion
*Patient assessment*	
If I upload pictures of myself, can you recommend to me which type of implants, size, and specific type of breast augmentation technique would be best for me?	Acknowledges limitations as an AI model with the inability to directly view or analyze images and provide personalized recommendations Nonetheless, provides patients with general guidelines, considerations, and technologies (3D imaging) plastic surgeons may use for surgical planning in this context
*Preparation for surgery*	
I am a (*x*)-year-old female, with (medical and lifestyle risk factors) … is there anything I can do to prepare for this surgery and make myself the best candidate?” If I decide to get (procedure), what can I expect on the lead-up to my surgery?	Capable of counseling patients on important lifestyle modifications (such as smoking and drug cessation), as well as optimization of medical comorbidities, with relevant physicians and healthcare providers, in preparation for surgery Encourages health lifestyle choices such as diet and exercise, open communication with surgeons, following of pre- and postsurgical instructions, arranging for support Capable of providing patients with a general outline of the surgical journey, from initial consultation to preoperative evaluation, devising a surgical plan, scheduling, preoperative optimization, consent, financial arrangements, and preparation for recovery
*Recovery and postprocedural instructions*	
What is the recovery process like regarding the ability to perform my normal day-to-day activities? How long will I need assistance for? How long before I can have intercourse? How long before I can go out in public? How long before I can return to work, and start working out? Will I still need to wear bras after (procedure)? What can I do to best-preserve my results, and what are things that can make my results worse that I should avoid?	Capable, within reasonable accuracy, of answering patient questions pertaining to the recovery period, and providing a general timeline to patients with an idea of what to expect with regards to symptoms, activity levels, and limitations during the first few days after surgery, first, 1 to 2, 4 to 6, and 6 to 8 weeks postoperatively Capable of answering specific questions, with reasonable yet imperfect accuracy, regarding required assistance with activities of daily living, intercourse, permitted activity levels/exercise, and return to work Capable of providing patients with expectations regarding the longevity of results, as well as some recommendations regarding optimal practices to best-preserve postoperative aesthetic outcomes, including requirements of supportive dressings and short/long-term bra use Varying accuracy and relevance of some recommendations (eg, sun protection and sunscreen to prevent hyperpigmentation of mastopexy scars)
*Procedure cost and surgeon recommendations*	
How much does (procedure) cost? Can it be covered by insurance? Can you recommend to me the best surgeon I can go to near my area?	Capable of providing patients with a general estimate on average procedure costs, while counseling them on the potential impact of a surgeon experience and expertise, geographic location, surgical facility, anesthesia fees, preoperative tests, postoperative care, and additional procedures or treatments required on overall price Shares with patients the general guidelines and limitations pertaining to insurance coverage, including the need for medically documented functional symptoms and potential requirement of preauthorization by insurance companies for breast reductions. Emphasizes that purely aesthetic augmentations or mastopexies are not usually covered unless deemed medically necessary (eg, balancing procedures) Fails to recommend specific surgeons to patients based on the suggestion of providing the model with the patient's geographic area Instead, provides patients with factors to consider when seeking to find a plastic surgeon for themselves, including board certification, experience and expertise, referrals and reviews, before-and-after photographs, credentials, hospital affiliations, and ability to forge a personal connection during consultations

General summary of answering capabilities presented. The wording of some questions are marginally altered for brevity and grouping. All original questions posed to ChatGPT and responses within each of the 3 simulated patient interactions are presented, in original form, within Supplementary Documents 1 to 3. 3D, 3-dimensional; BIA-ALCL, breast implant–associated anaplastic large cell lymphoma; BII, breast implant illness.

Four expert breast plastic surgeons (H.F., D.H., W.P.A., and F.N.) evaluated the responses ChatGPT provided for the 3 aforementioned procedures, noting any inaccuracies or missing information. An objective assessment of responses was performed using standardized Likert scales from 1 to 10 (10 representing perfect performance); the criteria examined included accuracy of responses, comprehensiveness, objectivity, safety, communication clarity, and acknowledgment of limitations. A postparticipation survey was administered to solicit the experts' impressions on the perceived utility of ChatGPT, anticipated applications, and limitations. The means and standard deviations were calculated for all responses, and analyses performed using Microsoft Excel (Microsoft Technology Inc., Redmond, WA). One-way analysis of variance (ANOVA) was used for statistical analysis and a *P*-value <.05 was taken to indicate significance.

## RESULTS

### Performance

Average time required for ChatGPT to provide the user with an answer to a posed question was 28.6 s, with an average response length of 360 words. When normalizing to the number of questions posed within each user input (in the case of questions with multiple subcomponents), average response time was 18.6 s per question, with an average response length of 230 words per question.

**Figure 1.**
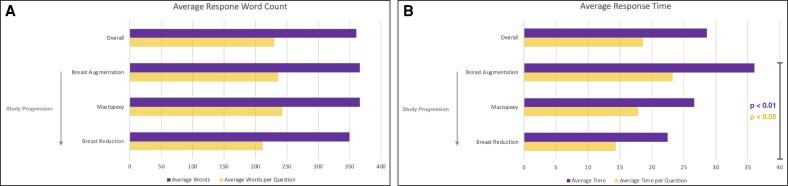
Average ChatGPT response word count and response time. (A) Visual depiction of the average response word counts provided by ChatGPT, overall and across all queries and procedures, and stratified according to the specific procedures examined. No significant differences were found in the average response word counts, before or after normalization to the numbers of questions posed within each query, as the study progressed. (B) Visual depiction of the average ChatGPT response time during the present study. Overall average response time (across all queries and all procedures), as well the overall average response time following normalization to the numbers of questions posed within each query, are presented. A statistically significant reduction in average response time was demonstrated for the standardized queries posed to ChatGPT as the study progressed, a possible reflection of the model's machine learning capabilities based on its history of interactions with users.

To assess the model's machine learning capabilities and the potential impact of previous exposure to the same set of standardized questions on future performance, response times and word counts were compared in paired sets across the order in which the simulated patient interactions with ChatGPT occurred, beginning initially with breast augmentation, then mastopexy, and finally breast reduction (Figure 1). No significant differences in word counts were found in this context (366 words, 366 words, and 349 words, respectively; *P* = .78). Notably, a statistically significant difference in time required for ChatGPT to provide the user with answers to the same set of standardized questions in subsequent user queries was significantly reduced (Figure 1). Average response times were 36, 26.6, and 22.5 s, respectively (*P* < .01, 1-way ANOVA). Average response times, per question in cases of questions with multiple subcomponents, were also significantly reduced (23.22 s per question, 17.9 s per question, and 14.4 s per question, respectively; *P* < .05, 1-way ANOVA).

### Assessment

ChatGPT provided an explicit disclaimer regarding its limitations as an AI model in answering medical questions, and/or encouraged patients to consult with a board-certified plastic surgeon, in 100% of answers provided. Despite being prompted to ask any questions it deems necessary to better-understand patients' needs and concerns, ChatGPT did not pose any questions to the user within these simulated interactions.

The overall performance across all evaluation metrics and question categories examined is presented in Table 2. The overall score, representing ChatGPT's performance across all question categories, assessment criteria, and aesthetic breast plastic surgery procedures examined was 7.3/10 ± 0.5. Overall, the accuracy of information was scored at 7.1/10 ± 0.5; comprehensiveness at 7.0/10 ± 0.6, objectivity at 7.5/10 ± 0.4, safety at 7.5/10 ± 0.4, communication clarity at 7.3/10 ± 0.2, and acknowledgment of limitations at 7.7/10 ± 0.2. With regards to specific procedures examined, ChatGPT's average score was 7.0/10 ± 0.8 for breast augmentation; 7.6/10 ± 0.5 for mastopexy, and 7.4/10 ± 0.5 for breast reduction. The performance on breast implant–specific knowledge was scored at 6.7/10 ± 0.6. A detailed breakdown of performance across the 8 standardized question categories, 6 assessment criteria employed, and for all 3 aesthetic breast plastic surgery procedures examined is presented in Tables 3 to 5. A summary of evaluators' responses to the survey evaluating their experience with ChatGPT, and perceived applications/limitations, is presented in Table 6.

**Table 2.** Overall Expert Panel Assessment Scores Across All Simulated Patient Inquiries With ChatGPT on Aesthetic Breast Plastic Surgical Procedures

Overall	Accuracy of information	Comprehensiveness	Objectivity of information	Safety of information shared with patient	Acknowledgment of limitations	Communication, clarity, patient-appropriate readability	Average
Aesthetic needs inquiry; awareness of available procedures	7.3 ± 0.0	6.8 ± 0.7	7.8 ± 0.7	7.4 ± 0.3	7.8 ± 0.3	7.5 ± 0.4	7.4 ± 0.4
Patient candidacy and procedure indications	7.1 ± 0.3	7.0 ± 0.7	7.2 ± 0.1	7.0 ± 0.4	7.8 ± 0.5	7.1 ± 0.5	7.2 ± 0.3
Procedure safety and risks	6.6 ± 0.8	6.4 ± 0.9	6.8 ± 0.5	6.9 ± 0.6	7.4 ± 0.6	6.9 ± 0.8	6.8 ± 0.3
Procedure information, steps, and techniques	7.2 ± 0.8	7.2 ± 1.0	7.3 ± 0.5	7.5 ± 0.4	7.6 ± 0.5	7.3 ± 0.4	7.3 ± 0.2
Patient assessment	6.1 ± 0.4	6.0 ± 0.3	7.8 ± 0.4	7.9 ± 0.3	7.9 ± 0.1	7.5 ± 0.3	7.2 ± 0.9
Preparation for surgery	7.6 ± 0.5	7.7 ± 0.4	7.8 ± 0.5	7.8 ± 0.5	7.8 ± 0.4	7.4 ± 0.6	7.7 ± 0.2
Recovery and postprocedure instructions	7.3 ± 0.5	7.3 ± 0.5	7.4 ± 0.5	7.4 ± 0.8	7.5 ± 0.4	7.1 ± 0.6	7.3 ± 0.2
Procedure cost and surgeon recommendations	7.6 ± 0.6	7.6 ± 0.6	7.8 ± 0.7	7.7 ± 0.5	7.8 ± 0.6	7.6 ± 0.4	7.7 ± 0.1
Average	7.1 ± 0.5	7.0 ± 0.6	7.5 ± 0.4	7.5 ± 0.4	7.7 ± 0.2	7.3 ± 0.2	7.3 ± 0.5

Means ± standard deviations presented.

**Table 3.** Expert Panel Assessment Scores on ChatGPT's Performance in the Simulated Patient Inquiry on Breast Augmentation

Breast augmentation	Accuracy of information	Comprehensiveness	Objectivity of information	Safety of information shared with patient	Acknowledgment of limitations	Communication, clarity, patient-appropriate readability	Average
Aesthetic needs inquiry; awareness of available procedures	7.3 ± 2.9	6.0 ± 1.8	8.5 ± 0.6	7.3 ± 2.9	7.5 ± 3.1	7.3 ± 2.4	7.3 ± 0.8
Patient candidacy and procedure indications	6.8 ± 3.2	6.3 ± 3.0	7.3 ± 2.9	6.5 ± 3.1	7.3 ± 3.6	6.5 ± 3.1	6.8 ± 0.4
Procedure safety and risks	5.8 ± 3.0	5.5 ± 3.7	6.3 ± 3.1	6.3 ± 3.1	6.8 ± 3.6	6.3 ± 3.0	6.1 ± 0.4
Procedure information, steps, and techniques	6.3 ± 3.6	6.0 ± 3.5	6.8 ± 3.3	7.0 ± 3.4	7.0 ± 4.1	7.0 ± 2.2	6.7 ± 0.4
Breast implant–specific knowledge	6.3 ± 3.6	5.8 ± 3.4	6.8 ± 3.3	7.0 ± 3.4	7.0 ± 4.1	7.3 ± 2.4	6.7 ± 0.6
Patient assessment	6.5 ± 3.8	6.3 ± 3.6	8.3 ± 1.0	8.3 ± 1.0	8.0 ± 2.2	7.8 ± 1.0	7.5 ± 0.9
Preparation for surgery	7.0 ± 2.8	7.3 ± 2.9	7.3 ± 2.9	7.3 ± 2.9	7.5 ± 3.1	6.8 ± 2.6	7.2 ± 0.3
Recovery and postprocedure instructions	6.8 ± 3.2	6.8 ± 3.2	7.0 ± 3.4	6.8 ± 3.2	7.3 ± 3.6	6.5 ± 3.1	6.8 ± 0.3
Procedure cost and surgeon recommendations	8.3 ± 1.0	8.3 ± 1.0	8.5 ± 1.0	8.3 ± 1.0	8.5 ± 1.3	8.0 ± 1.2	8.3 ± 0.2
Average	6.8 ± 0.7	6.4 ± 0.9	7.4 ± 0.8	7.2 ± 0.7	7.4 ± 0.5	7.0 ± 0.6	7.0 ± 0.8

Means ± standard deviations presented.

**Table 4.** Expert Panel Assessment Scores on ChatGPT's Performance in the Simulated Patient Inquiry on Mastopexy

Mastopexy	Accuracy of information	Comprehensiveness	Objectivity of information	Safety of information shared with patient	Acknowledgment of limitations	Communication, clarity, patient-appropriate readability	Average
Aesthetic needs inquiry; awareness of available procedures	7.3 ± 3.1	7.3 ± 3.1	7.5 ± 2.6	7.8 ± 2.2	8.0 ± 3.4	8.0 ± 1.8	7.6 ± 0.3
Patient candidacy and procedure indications	7.3 ± 3.0	7.3 ± 3.0	7.0 ± 2.9	7.3 ± 3.1	8.0 ± 3.4	7.3 ± 3.1	7.3 ± 0.3
Procedure safety and risks	7.3 ± 3.0	7.3 ± 3.0	7.3 ± 3.0	7.5 ± 3.1	8.0 ± 3.4	7.8 ± 2.2	7.5 ± 0.3
Procedure information, steps, and techniques	7.5 ± 3.1	7.8 ± 3.2	7.8 ± 3.2	7.8 ± 3.2	8.0 ± 3.4	7.8 ± 2.2	7.8 ± 0.2
Patient assessment	5.8 ± 4.4	5.8 ± 4.4	7.5 ± 3.1	7.8 ± 3.2	8.0 ± 3.4	7.3 ± 3.1	7.0 ± 1.0
Preparation for surgery	7.8 ± 4.1	7.8 ± 2.1	8.0 ± 2.2	8.0 ± 2.2	7.8 ± 2.1	7.5 ± 2.1	7.8 ± 0.2
Recovery and postprocedure instructions	7.8 ± 1.7	7.8 ± 1.7	8.0 ± 1.6	8.3 ± 1.7	8.0 ± 1.6	7.8 ± 1.7	7.9 ± 0.2
Procedure cost and surgeon recommendations	7.5 ± 2.1	7.5 ± 2.1	7.5 ± 2.1	7.5 ± 2.1	7.5 ± 2.1	7.5 ± 2.1	7.5 ± 0.0
Average	7.3 ± 0.6	7.3 ± 0.7	7.6 ± 0.3	7.7 ± 0.3	7.9 ± 0.2	7.6 ± 0.3	7.6 ± 0.5

Means ± standard deviations presented.

**Table 5.** Expert Panel Assessment Scores on ChatGPT's Performance in the Simulated Patient Inquiry on Breast Reduction

Breast reduction	Accuracy of information	Comprehensiveness	Objectivity of information	Safety of information shared with patient	Acknowledgment of limitations	Communication, clarity, patient-appropriate readability	Average
Aesthetic needs inquiry; awareness of available procedures	7.3 ± 3.1	7.3 ± 3.1	7.3 ± 3.1	7.3 ± 3.1	8.0 ± 3.4	7.3 ± 3.1	7.4 ± 0.3
Patient candidacy and procedure indications	7.3 ± 3.0	7.5 ± 2.5	7.3 ± 2.5	7.3 ± 2.5	8.3 ± 2.9	7.5 ± 2.6	7.5 ± 0.4
Procedure safety and risks	6.8 ± 3.0	6.5 ± 3.1	7.0 ± 2.9	7.0 ± 2.9	7.5 ± 3.3	6.8 ± 2.9	6.9 ± 0.3
Procedure information, steps, and techniques	7.8 ± 2.6	7.8 ± 2.6	7.5 ± 3.1	7.8 ± 3.2	7.8 ± 3.3	7.3 ± 3.1	7.6 ± 0.2
Patient assessment	6.0 ± 3.9	6.0 ± 3.9	7.8 ± 2.2	7.8 ± 2.1	7.8 ± 2.1	7.5 ± 2.1	7.1 ± 0.9
Preparation for surgery	8.0 ± 1.4	8.0 ± 1.4	8.3 ± 1.5	8.3 ± 1.3	8.3 ± 1.3	8.0 ± 1.4	8.1 ± 0.1
Recovery and postprocedure instructions	7.3 ± 3.0	7.3 ± 3.0	7.3 ± 3.0	7.3 ± 3.0	7.3 ± 3.0	7.0 ± 2.9	7.2 ± 0.1
Procedure cost and surgeon recommendations	7.0 ± 2.9	7.0 ± 2.9	7.3 ± 3.0	7.3 ± 3.0	7.5 ± 3.1	7.3 ± 3.1	7.2 ± 0.2
Average	7.2 ± 0.6	7.2 ± 0.7	7.4 ± 0.4	7.5 ± 0.4	7.8 ± 0.4	7.3 ± 0.4	7.4 ± 0.5

Means ± standard deviations presented.

**Table 6.** Postparticipation Survey

	Score
*Evaluation experience*	
Do you see value in the information ChatGPT is able to provide patients?	8.0 ± 3.4/10
Did you feel the level of communication and complexity of answers was appropriate for patients?	5.8 ± 3.4/10
Do you feel it is a safe alternative to patients who would be looking information up on Google on their own, or social media?	7.3 ± 1.9/10
Do you feel it is a safe alternative to administrative staff in a clinic answering patient questions?	6.3 ± 4.5/10
Do you feel it is a safe alternative to a nurse or physician assistant responding to patient questions?	6.5 ± 4.0/10
Do you feel more studies and/or societal guidelines are required for you to more accurately determine your views on its safety and utility?	5.8 ± 4.3/10
*Perceived applications*	
Do you believe the benefits outweigh risks associated with incorporating ChatGPT, in its current form, into your own practice?	6.0 ± 4.7/10
Are you open to incorporating ChatGPT into you practice in its future iterations, with more research and development?	8.8 ± 2.5/10
If you answered yes to any of the above questions, at what level can you see yourself incorporating ChatGPT into your practice? Please select all that apply:	
Patient education and guidance leading up to consultation	3/4, 75%
Patient selection for consultation	3/4, 75%
Postoperative patient care and counseling	2/4, 50%
Other Frequently asked questions (*n* = 1) Marketing and promotions (*n* = 1)	

Expert evaluator survey assessing overall impression of ChatGPT and perceived utility for its role in patient guidance and patient education.

## DISCUSSION

The present study evaluates the performance of AI technology with relevance to its potential application as a patient guidance and education resource, using aesthetic breast plastic surgery as a proof of concept. ChatGPT demonstrated an impressive overall performance, with an overall evaluation score of 7.3/10 ± 0.5 as assessed by 4 expert aesthetic breast plastic surgeons. Reasonable degrees of accuracy, comprehensiveness, clarity, safety, and acknowledgment of limitations were observed. The model proved safe and reliable by consistently emphasizing the need to consult with board-certified plastic surgeons. It demonstrated improved efficiency and enhanced performance as a possible testament to its machine learning capabilities. The authors concede, however, that despite this impressive performance, and although it could educate patients in the lead-up to their surgical consultations, it cannot replace the need for direct counseling and education by board-certified plastic surgeons.

### Aesthetic Needs Inquiry; Awareness of Available Procedures

ChatGPT proved capable of capturing and synthesizing patient-reported aesthetic needs, and subsequently presenting and contrasting different surgical options, albeit imperfectly. For example, when providing fat grafting as a potential option to Patient 1 for breast augmentation, fat survival and the likely need for multiple surgeries to achieve a meaningful result was undisclosed. In other examples, it overlooked the possibility of necessary concomitant procedures; for example, the need for additional breast work (and scar burden) in patients with tuberous breasts or significant ptosis. Some combination procedures suggested were also misleading, such as a “combination of breast lift and breast reduction.” Nonetheless, it consistently emphasized the need to consult with a board-certified plastic surgeon who could more appropriately assess the patients' individual needs, review the medical history, perform a physical examination, and discuss potential risks, recovery, and expected results.

### Patient Candidacy and Procedure Indications

In response to simulated patient profiles with different lifestyle and medical risk factors, ChatGPT generally proved capable of identifying and counseling patients on risk factors that may alter their candidacy for surgery. However, its ability to emphasize the relative impact of specific risk factors proved limited. For the simulated patient interested in breast reduction reporting recreational smoking and cocaine use, the bot counseled her on the general negative effects of these lifestyle choices on systemic and cardiovascular health, but failed to accentuate their relevance as absolute contraindications to this surgery. Similarly, it seemed to imply that a diagnosis of Attention Deficit Hyperactivity Disorder may have more of an impact on surgical planning than Crohn's disease and its medications, which are known to affect wound healing.^22^ Despite explicitly being prompted to ask the user any questions it deemed necessary to provide more personalized answers, ChatGPT failed to do so across all 3 simulated user interactions. Within this particular assessment category, a more thorough understanding of patients' needs could lead to more patient-appropriate recommendations, such as in deciding on the timing of a mastopexy before completing family planning. Soliciting information regarding the impact of a patient's ptosis on her physical, emotional, and sexual wellbeing, relative to the importance and likelihood of having future children, should be considered. Nevertheless, the bot encouraged patients to disclose all information to their plastic surgeon, to whom it generally deferred for decisive assessment and conclusions on whether patients examined were good candidates for surgery.

### Procedure Safety and Risks

ChatGPT proved capable of providing a comprehensive yet incomplete list of possible complications associated with different surgical procedures. Inconsistent in its ability to provide specific estimates of incidence, it shared specific estimates for complications associated with breast augmentation, but not for mastopexy or breast reduction. In the latter cases, the order in which it listed complications could be challenged, as chronology could indirectly imply likelihood. T-junction necrosis and dehiscence should be discussed before infection in breast reduction, for example. Nonetheless, ChatGPT also proved capable of discussing implications associated with certain complications (such as implant loss in severe infections).

Nuances and specific details shared within discussions of risks and complications are of paramount importance, both from the medico-legal and ethical perspectives. In the present study, several omissions of notable significance were identified. When queried about risks associated with breast augmentation, risks such as reoperation, implant malposition, breast implant illness (BII), and breast implant–associated anaplastic large cell lymphoma (BIA-ALCL) were not discussed. The burden subsequently fell on the simulated patient to inquire about any additional “long-term” complications, following which some, but not all, were disclosed. The bot also missed several opportunities to expand on relevant-related risks, such as discussing the risk of BIA-ALCL and capsular squamous cell carcinoma when a patient inquired about whether breast implants cause breast cancer. This is especially relevant in recent times, as the narrative for BII and BIA-ALCL is becoming increasingly written by social media, online forums, and chatrooms, rather than scientific evidence.^23-26^ It would be interesting to speculate on whether ChatGPT could capture misinformation found on social media, and instead use data from peer-reviewed journals to better inform patients on BII and BIA-ALCL.

Expectation management remains an essential aspect of aesthetic surgical care delivery, and specific nuances in word choices, and implied connotations, can be of great significance for informed consent and patient satisfaction. In several instances, ChatGPT's word choices were deemed suboptimal, potentially misleading patients and impacting expectations. Statements such as “perfect symmetry cannot *always* be achieved,” for example, incorrectly imply that there exist cases in which it could. Finally, the ability of ChatGPT to qualify statements and recommendations it makes in relation to varying levels of available evidence remains unclear. For example, and while there exist some studies reporting on marginally decreased breastfeeding abilities following breast augmentation,^27,28^ the available evidence remains inconclusive and incongruent with the wording choices used by ChatGPT, specifically with regards to the relative impact of subglandular vs subpectoral implant pockets.

### Procedure Information, Steps, Technique, and Breast Implant Information

ChatGPT proved capable of providing patients with a general and structured outline of surgical steps at a patient-appropriate level. Elements discussed included anesthesia and its possibilities (general vs sedation), incision placement options, breast implant pocket considerations, implant options, tissue removal, reshaping, nipple repositioning in mastopexy/breast reduction, as well as incision closure and dressings. A limited discussion of different techniques, within each procedure, was also provided (eg, wise-pattern vs circumvertical vs circumareolar lifts), although no discussion of more advanced topics, such as pedicle choice, was presented. With relevance to breast implants, ChatGPT proved capable of providing a thorough yet incomplete comparative primer with relevance to fill, texture, plane of insertion, and incision/approach. Several critical omissions were also noted, from nondisclosure of certain techniques (such as subfascial augmentations), to the association of certain surgical choices with postoperative outcomes such as animation deformity with subpectoral pockets, and lower capsular contracture rates with inframammary approaches.

### Patient Assessment

ChatGPT consistently acknowledged its limitations and inability to analyze images provided by patients for personalized recommendations. Nonetheless, it provided general guidelines and considerations plastic surgeons may use for surgical planning, including patient-desired outcome, body proportions, tissue characteristics, and overall health. Omitted, however, were discussions on limitations patients' breast dimensions and asymmetry may present.

### Preparation for Surgery

ChatGPT counseled patients on important lifestyle modifications such as smoking and drug cessation, as well as optimization of medical comorbidities before surgery. It encouraged healthy lifestyle choices such as diet and exercise, open communication with surgeons, arranging of support, and following of pre- and postsurgical instructions. It provided patients with an overview of what the journey toward surgery may entail from initial consultation, to preoperative evaluation and optimization, generation of a surgical plan, consent, scheduling, financial arrangements, and preparation for recovery.

### Recovery and Postprocedure Instructions

Within reasonable accuracy, ChatGPT provided simulated patients with answers to questions about the recovery period, including expected symptom and activity limitations within the first, 1 to 2, 4 to 6, and 6 to 8 weeks. It proved capable of answering specific questions, within reasonable accuracy, regarding assistance needed with activities of daily living, permitted activity levels, intercourse, exercise, and return to work. Some guidelines diverged from accepted standards; although ChatGPT noted that exercise may be started at 4 to 6 weeks following breast augmentation, some surgeons may prefer that high impact exercise be started no earlier than 6 weeks. It also overestimated the time before which a patient with a mastopexy can likely go out in public; incorrectly indicating 2 weeks. The authors find that 1 week is often sufficient, with some patients capable of leaving the house within 1 to 2 days. Some recommendations were odd and misplaced, such as emphasis on avoiding sun exposure for optimal scar cosmesis in mastopexy, despite breasts being generally photograph protected, whereas other recommendations were not qualified by the greatest evidence, such as the need for long-term supportive bra use following breast reductions.

### Procedure Cost and Surgeon Recommendations

ChatGPT was capable of providing patients with general estimates on average procedure costs, while accentuating the potential impact of surgeon experience, expertise, geographic location, facility, anesthesia fees, preoperative tests, postoperative care, and additional procedures or treatments on overall price. ChatGPT also shared general guidelines and limitations relating to insurance coverage, including the need for medically documented functional symptoms in breast reductions, and that purely aesthetic augmentations or mastopexies are not usually covered. It failed to recommend specific surgeons to patients based on their geographic location; instead, it provided patients with factors to consider when seeking one themselves. These included board certification, experience and expertise, referrals, reviews, before-and-after photographs, credentials, hospital affiliations, and ability to forge a personal connection. Although it provided the American Society of Plastic Surgeons and Aesthetic Society websites as reference resources in this context, it is noteworthy that it referred to the latter as the American Society for Aesthetic Plastic Surgery, which may potentially confuse patients.

### Wording and Language Choice Limitations

Overall, the writing style used by ChatGPT was found to be excessively dry, verbose, and overly inclusive. This may be unsurprising, given that ChatGPT is trained on vast amounts of readily available data from the Internet, likely filled with empty buzzwords. With an average response length of 360 words, there exists room for optimization with respect to reading level and brevity. Less time invested by patients laboring to read through wordy responses may allow for more exchanges between patient and bot, and thus, potentially more patient-specific responses and recommendations. Patients who are technologically savvy can ask ChatGPT to explain responses on a specific reading level and truncate responses to a specific word limit. Choices in wording can also be of great significance in managing patient expectations. The stakes to relinquish control of information shared with patients is of great significance. Any inaccuracies or suboptimal word choices could, at minimum, mislead patients and alter expectations, or more concerningly, compromise the integrity of the patient–physician relationship and culminate in potential lawsuits.

### Applications and Future Directions

The present study demonstrates that AI LLM technology represents a promising resource for patient education, although currently available AI models may not be used in their native forms to safely and accurately educate patients. Adoption of AI technology for this application would therefore begin by fine-tuning and further training these models with evidence-based data and information, selected and curated by plastic surgeons, and/or our professional societies. This would provide patients with an interactive educational reference, in contrast to traditional resources such as pamphlets and websites, that can help patients become better-informed while responding to their evolving educational needs and new technologies.

### Study Limitations

The limitation of the present study is that only 4 expert evaluators were used to assess the performance of ChatGPT. Additionally, LLMs are amalgams of what they retrieve from the Internet at any given time; it is possible that responses may change given the fluidity of the Internet. Although this study provides generalizability for the average patient seeking aesthetic breast surgery, it is unclear whether these responses can and will be replicated with exact prompts given in different situations. One interesting finding is that despite deleting chats, resetting browser data, and restarting the computer, the bot's response times became progressively shorter after each patient profile. Presumably, each independent chat should represent a new neural network; however, this does not seem to be the case. This finding only reinforces the fact that there remains much that is unknown about AI and ChatGPT.

## CONCLUSIONS

The present study demonstrates that AI LLM technology may represent helpful patient educational resources in the lead-up to the surgical consultation, but cannot replace direct counseling by plastic surgeons. Although the information was neither entirely accurate nor comprehensive, there were no unsafe recommendations provided. The bot encourages consultation with board certified plastic surgeons in all of its responses. Significant limitations persist with any technology that falls short, in any aspect of performance, relative to the highest standards of care. Limitations encountered may be best addressed by seeking to further train and modify the models, by influencing the resources on which they are trained.

## Supplementary Material

ojae062_Supplementary_Data
