# Supplementary material for: Preoperative Patient Guidance and Education in Aesthetic Breast Plastic Surgery: A Novel Proposed Application of Artificial Intelligence Large Language Models
Source: Aesthet Surg J Open Forum. 2024 Aug 13;6:ojae062. doi: 10.1093/asjof/ojae062 (PMC11385898; doi:10.1093/asjof/ojae062)
Supplement: ojae062_Supplementary_Data [file ojae062_supplementary_data.zip › Appendix A.pdf]

30.46s

Category 1:

Aesthetic Needs Inquiry; Awareness of Available Procedures

I have some questions about breast plastic surgery I'd like to ask you. Feel free to ask me more questions if you need, so you can to give me the best answers. I'm bothered by the size and shape of my small breasts, and I think I want to get plastic surgery to make them bigger. What are my options?

I understand that you're considering breast plastic surgery to enhance the size and shape of your breasts. There are several options available for breast augmentation, and it's important to consult with a qualified plastic surgeon to discuss your specific goals and concerns. However, I can provide you with some general information about the common options:

1. Breast Implants: This is the most popular method for breast augmentation. Implants are silicone shells filled with either saline solution or silicone gel. They are inserted into the breasts to increase their size and enhance their shape. There are different types of implants available, and your surgeon can guide you in choosing the right size, shape, and material based on your preferences and anatomy.
2. Fat Transfer: This procedure involves removing fat from one part of your body, such as the abdomen or thighs, through liposuction, and then injecting it into your breasts to increase their volume. Fat transfer provides a natural look and feel, but the degree of enhancement may be limited compared to implants.
3. Combination Approach: In some cases, a combination of breast implants and fat transfer can be used to achieve the desired outcome. This approach allows for a more customized result, as the surgeon can utilize implants for volume and shape while using fat transfer to refine the contours and provide additional fullness.

During your consultation with a plastic surgeon, they will evaluate your breast anatomy, discuss your goals, and help you choose the most suitable option based on factors such as your body type, desired size, and personal preferences. It's important to have realistic expectations and ensure that you fully understand the potential risks and benefits of the procedure before making a decision.

24.93s

Category 2:

Patient Candidacy and Procedure Indications

I'm a 27-year-old-female, drink occasionally, and vape daily. I have ADHD and take medications for it, and well-controlled Chron's disease on medications. Am I a good candidate for this surgery?

I'm not a doctor, but I can provide some general information. Ultimately, it's important to consult with a qualified plastic surgeon who can assess your specific medical history and determine if you are a good candidate for breast plastic surgery.

In general, being in good overall health is important for any surgical procedure, including breast augmentation. Here are some factors to consider:

1. Medical Conditions: Having well-controlled Crohn's disease and taking medications for it is a positive factor. However, it's essential to disclose your medical history, including ADHD and any other medical conditions, to your plastic surgeon. They can evaluate how these conditions may impact the surgery and your recovery.
2. Medications: Inform your surgeon about any medications you're taking, including those for ADHD. Some medications may have interactions or potential effects on the surgery and anesthesia. Your surgeon will assess whether your current medication regimen is compatible with the procedure.
3. Lifestyle Choices: Daily vaping and occasional drinking may have an impact on your candidacy for surgery. Nicotine use, including vaping, can affect wound healing and increase the risk of complications. It's crucial to be honest with your surgeon about your habits, as they may provide recommendations to minimize potential risks.

During your consultation, your plastic surgeon will evaluate your medical history, conduct a physical examination, and discuss your goals and expectations. They will determine if you are a suitable candidate for the surgery based on your unique circumstances.

Remember, being open and honest with your surgeon about your medical history, lifestyle choices, and expectations is crucial for ensuring a safe and successful surgical experience.

Category 3:

Procedure Safety and Risks

42.37s

What are the risks associated with breast augmentation? Can you give me specific estimates, and an overall complication rate?

Breast augmentation, like any surgical procedure, carries certain risks and potential complications. It's important to understand these risks and discuss them in detail with your plastic surgeon. While I can provide you with a general overview, it's essential to consult with a qualified medical professional for personalized information. Keep in mind that specific estimates and complication rates can vary depending on various factors, including the surgical technique, implants used, surgeon's experience, and individual patient characteristics.

Here are some potential risks and complications associated with breast augmentation:

1. Infection: The risk of infection is relatively low, estimated to be around 1-2%. However, if an infection occurs, it may require antibiotics or, in severe cases, implant removal.
2. Capsular Contracture: This refers to the formation of excessive scar tissue around the implant, causing the breasts to feel firm and potentially altering their appearance. The incidence of capsular contracture varies but is generally estimated to be around 5-10%.
3. Implant Rupture or Leakage: Implants can rupture or leak over time. The risk of rupture depends on the type of implant and can range from less than 1% to around 10% over ten years. Saline implants deflate when ruptured, while silicone gel implants may have silent ruptures that are only detectable through imaging.
4. Changes in Sensation: Temporary or permanent changes in nipple or breast sensation may occur, though significant sensory changes are relatively rare.
5. Hematoma and Seroma: These are collections of blood or fluid that can occur around the implant or in the surgical area. They may require drainage.
6. Scarring: Breast augmentation involves incisions, resulting in scars. The extent and visibility of scars depend on various factors, including incision type and individual healing.
7. Asymmetry: Perfect symmetry cannot always be achieved, and there may be slight differences in the appearance of the breasts following surgery.

It's worth noting that advances in surgical techniques and implant technology have helped to minimize these risks, and the majority of breast augmentations are successful with satisfying outcomes. The overall complication rate can vary, but studies generally report complication rates ranging from 10-25%.

Your plastic surgeon will provide you with a comprehensive understanding of the risks and complications specific to your case during your consultation. They will also take into account your medical history, lifestyle factors, and individual circumstances to give you a more accurate assessment of the potential risks you may face.

Category 3:

Procedure Safety and Risks

41.78s

Are there long-term complications I should know about regarding breast augmentation and implants, and can you give me specific estimates?

Breast augmentation with implants can have long-term complications, although they are generally uncommon. It's important to have a thorough discussion with your plastic surgeon about the potential risks and long-term effects specific to your situation. While I can provide general information, specific estimates may vary based on individual factors and the type of implant used.

Here are some long-term complications associated with breast augmentation:

1. Capsular Contracture: Capsular contracture can occur years after surgery. It happens when the scar tissue around the implant tightens, causing the breast to feel firm, distorted, or uncomfortable. The incidence of capsular contracture varies, but long-term rates have been reported to be around 15-20% over 10 years.
2. Implant Rupture or Leakage: Implants can rupture or leak over time, although the exact rate depends on the type of implant. For silicone gel implants, the risk of silent rupture increases over time. Studies have estimated the rupture rate for silicone gel implants to be around 10% or less over ten years, while saline implants have a lower rupture rate.
3. Implant Displacement or Malposition: In some cases, the implants may shift or move from their original position over time. This can result in asymmetry or an unnatural appearance of the breasts. The risk of implant displacement can be influenced by factors such as implant placement, surgical technique, and anatomical characteristics.
4. Changes in Breast Tissue: Breast implants can affect the way breast tissue looks and feels during mammography screenings. It's important to inform the radiologist about your implants to ensure accurate interpretation of the results. Routine mammograms can still be performed, although additional imaging techniques such as MRI may be recommended in some cases.
5. Breast Implant-Associated Anaplastic Large Cell Lymphoma (BIA-ALCL): BIA-ALCL is a rare type of lymphoma associated with textured surface breast implants. It is important to note that BIA-ALCL is extremely rare, and the risk is primarily associated with textured implants rather than smooth implants. Estimates of the risk range from 1 in 3,817 to 1 in 30,000 patients with textured implants, but this can vary based on various factors.

It's crucial to regularly monitor your breast health and follow up with your plastic surgeon for routine check-ups and any concerns that may arise. Your surgeon can provide you with more specific estimates of long-term complications based on your implant type, surgical technique, and individual characteristics.

Category 3:

30.24s

Procedure Safety and Risks

I have a few more questions about risks. Can I breastfeed after a breast augmentation? Will breast augmentation increase my chances of getting or detecting breast cancer in the future?

Here are answers to your questions about risks and considerations related to breast augmentation:

1. Breastfeeding: Breast augmentation can potentially affect breastfeeding, but the impact can vary depending on various factors, such as the surgical technique used and implant placement. Incisions made around the areola (periareolar incision) may carry a higher risk of interfering with milk ducts and breastfeeding compared to other incision options. Implants placed underneath the chest muscle (submuscular placement) may have a lower impact on breastfeeding compared to implants placed above the muscle (subglandular placement). It's important to discuss your desire to breastfeed in the future with your plastic surgeon during the consultation so they can plan the procedure accordingly and provide guidance based on your individual circumstances.
2. Breast Cancer and Detection: Breast augmentation itself does not increase the risk of developing breast cancer. However, it's important to note that breast implants may affect breast cancer screening and detection. Breast implants can potentially interfere with mammography, making it more challenging to visualize breast tissue. It's crucial to inform your radiologist about your implants before undergoing a mammogram to ensure they can take appropriate measures to obtain accurate results. Additional imaging techniques, such as ultrasound or MRI, may be recommended in some cases. Regular breast self-examination and routine breast cancer screenings, as recommended by healthcare professionals, remain important even after breast augmentation.

It's important to have a thorough discussion with your plastic surgeon about these concerns during your consultation. They can provide you with personalized information based on your specific circumstances and address any additional questions or concerns you may have. Additionally, consulting with your primary care physician or a breast specialist can also provide further guidance regarding breastfeeding and breast cancer screening after breast augmentation.

Category 3:

31.76s

Procedure Safety and Risks

Is it safe to combine breast augmentation with other procedures?

Combining breast augmentation with other procedures can be safe and is commonly done, depending on your overall health, the extent of the procedures, and other individual factors. However, it's essential to consult with a qualified plastic surgeon who can evaluate your specific case and provide personalized recommendations.

Combining procedures can offer several advantages, including a single surgical session, reduced overall recovery time, and potentially lower costs compared to undergoing multiple surgeries separately. It can also help achieve a more comprehensive and balanced aesthetic outcome when multiple areas of concern are addressed simultaneously.

Common procedures that are often combined with breast augmentation include:

1. Breast Lift (Mastopexy): If you have sagging or ptotic breasts in addition to desiring increased size or volume, a breast lift can be performed in conjunction with breast augmentation. This combination can enhance breast shape, position the nipples properly, and provide fullness.
2. Liposuction: Liposuction is frequently combined with breast augmentation to improve body contours by removing excess fat from areas such as the abdomen, flanks, or thighs. Combining liposuction with breast augmentation can provide more comprehensive body reshaping.
3. Tummy Tuck (Abdominoplasty): Women who desire breast augmentation and also have excess abdominal skin and muscle laxity may choose to undergo a tummy tuck in combination with breast surgery. This combination addresses both the breasts and abdomen, providing a more harmonious result.

The decision to combine procedures depends on various factors, including your overall health, the extent of surgery, the time required for anesthesia, and the potential risks involved. Your plastic surgeon will evaluate your medical history, perform a physical examination, and discuss your goals to determine if combining procedures is safe and appropriate for you.

It's crucial to have a thorough discussion with your plastic surgeon, understand the potential risks and benefits, and have realistic expectations regarding the recovery process and outcomes of combining procedures.

Category 4:

Procedure Information, Steps, & Techniques

45.96s

Can you explain to me the steps of how a breast augmentation is performed?

Certainly! While the specific steps of a breast augmentation procedure can vary based on individual factors and surgical techniques, here is a general outline of the process:

1. Anesthesia: The surgery begins with the administration of anesthesia. You and your surgeon will have previously discussed the type of anesthesia to be used, which may be general anesthesia (where you are unconscious) or intravenous sedation with local anesthesia (where you are sedated but awake).
2. Incision Placement: Your surgeon will make an incision, the location of which depends on factors such as implant type, desired outcome, and your anatomy. Common incision options include:
   - Inframammary Incision: This incision is made in the crease where the breast meets the chest wall.
   - Periareolar Incision: The incision is made along the lower border of the areola (the darker area surrounding the nipple).
   - Transaxillary Incision: The incision is made in the armpit, and the implant is inserted through a tunnel to the breast.
3. Implant Placement: Your surgeon will then create a pocket where the implant will be placed. There are two main options for implant placement:
   - Subglandular Placement: The implant is positioned between the breast tissue and the chest muscle.
   - Submuscular Placement: The implant is placed beneath the chest muscle (pectoralis major), providing additional coverage.
4. Insertion of the Implant: The chosen implant, either saline or silicone gel-filled, is carefully inserted into the pocket through the incision. The surgeon will ensure proper positioning and symmetry.
5. Incision Closure: Once the implant is in place, your surgeon will close the incisions with sutures, skin adhesive, or surgical tape. The incision closure method can vary depending on the surgeon's preference and the type of incision made.
6. Recovery and Post-Operative Care: After the surgery, you will be moved to a recovery area where you will be monitored until the effects of anesthesia wear off. You may be provided with a supportive bra or bandage to wear to aid in healing and proper implant positioning. Your surgeon will provide specific post-operative instructions regarding pain management, activity restrictions, and follow-up appointments.

It's important to note that this is a general overview, and the exact details of the procedure may vary based on your surgeon's approach, your unique anatomy, and the specific choices made during your consultation.

During your consultation, your plastic surgeon will discuss the details of the procedure, including incision options, implant placement, and any additional considerations based on your goals and individual characteristics. They will guide you through the process and ensure that you have a comprehensive understanding of what to expect.

Category 4:

44.56s

Procedure Information, Steps, & Techniques

(Includes Implant-Specific Knowledge)

You mentioned different kinds of implants, places of incision placement, and pockets where the implants will go. Can I choose what I want? Can you tell me the advantages and disadvantages of each?

Certainly! As a patient, you can have input and make choices regarding the type of implant, incision placement, and implant pocket during your breast augmentation procedure. Here are the advantages and disadvantages of each option:

Implant Types:

1. Saline Implants:
   - Advantages:
     - Can be inserted while empty, resulting in smaller incisions.
     - Saline solution is harmless and easily absorbed by the body in case of implant rupture.
   - Disadvantages:
     - May not feel as natural as silicone gel implants.
     - Greater risk of visible rippling or wrinkling.
2. Silicone Gel Implants:
   - Advantages:
     - Often considered to have a more natural look and feel compared to saline implants.
     - Less likely to experience visible rippling.
   - Disadvantages:
     - Requires a slightly longer incision for placement.
     - Silent rupture may be undetectable without imaging (regular monitoring recommended).
     - If a silicone gel implant ruptures, the gel can stay within the implant pocket or, in rare cases, migrate to nearby areas.

Incision Placement:

1. Inframammary Incision:
   - Advantages:
     - Provides direct access and good visibility for precise implant placement.
     - Incision location is concealed within the breast crease.
   - Disadvantages:
     - Potential for visible scarring, though typically well-hidden within the natural breast crease.
2. Periareolar Incision:
   - Advantages:
     - Incision is made along the lower border of the areola, allowing for potential scar camouflage.
     - Provides direct access and good visibility for implant placement.
   - Disadvantages:
     - Increased risk of altered nipple sensation.
     - Potential for more difficulty with breastfeeding compared to other incision options.
3. Transaxillary Incision:
   - Advantages:
     - No incisions on the breast itself, which may be desirable for some patients.
     - Potential for less visible scarring on the breast.
   - Disadvantages:
     - Requires a longer tunneling approach for implant placement.
     - Limited visibility during surgery, which can make precise pocket creation more challenging.

Implant Pocket:

1. Subglandular Placement:
   - Advantages:
     - Typically involves a quicker recovery and less discomfort compared to submuscular placement.
     - May provide more fullness in the upper pole of the breast.
   - Disadvantages:
     - Potentially higher risk of visible rippling, especially in individuals with thin breast tissue.
     - Slightly higher risk of capsular contracture (scar tissue formation around the implant).
2. Submuscular Placement:
   - Advantages:
     - Provides more tissue coverage over the implant, reducing the risk of visible rippling.
     - Potentially interferes less with mammography.
   - Disadvantages:
     - Involves a longer recovery period and may cause temporary muscle-related discomfort.
     - May result in a slightly flatter appearance in the upper pole of the breast.

It's important to discuss these options and their specific advantages and disadvantages with your plastic surgeon. They will evaluate your individual anatomy, consider your goals, and provide recommendations based on their expertise. Together, you can make informed decisions that align with your desired outcome and preferences.

Category 4:

35.93s

Procedure Information, Steps, & Techniques

(Includes Implant-Specific Knowledge)

I want to know a bit more about breast implants. Are they considered to be safe? Do they cause any diseases or cancer?

Breast implants have undergone extensive testing and research, and they are generally considered to be safe and effective medical devices. Both saline and silicone gel breast implants have been approved by regulatory authorities, such as the U.S. Food and Drug Administration (FDA), for use in breast augmentation and breast reconstruction surgeries.

However, it's important to be aware of potential risks and complications associated with breast implants. These can include:

1. Capsular Contracture: This occurs when scar tissue forms around the implant, causing the breast to feel firm or hard. It may require additional treatment or implant removal.
2. Implant Rupture: Implants can rupture or leak, which may require surgical intervention to remove or replace the implant.
3. Infection: Infection is a potential risk with any surgical procedure. In some cases, it may require implant removal until the infection is resolved.
4. Changes in Sensation: Temporary or permanent changes in nipple or breast sensation can occur after breast augmentation surgery.
5. Changes in Breastfeeding: Breast augmentation can potentially impact breastfeeding, especially with certain incision and implant placement techniques. Discussing your desire to breastfeed with your surgeon is important.

Regarding the association with diseases and cancer, research has not shown a causal relationship between breast implants and systemic diseases or an increased risk of breast cancer. However, there are specific considerations:

1. Breast Implant-Associated Anaplastic Large Cell Lymphoma (BIA-ALCL): BIA-ALCL is a rare type of lymphoma that has been associated with textured breast implants. It is not breast cancer but rather a cancer of the immune system. The risk of BIA-ALCL is considered low, and it primarily affects those with textured implants. Regular follow-up and monitoring are recommended to detect any potential signs or symptoms.
2. Breast Cancer Detection: Breast implants can make it more challenging to detect breast cancer during mammography. Additional imaging techniques, such as ultrasound or MRI, may be recommended to aid in breast cancer screening.

To ensure the safety and efficacy of breast implants, it's crucial to choose a qualified and experienced plastic surgeon, follow post-operative care instructions, and attend regular follow-up appointments. It's also important to stay informed about the latest research and consult with healthcare professionals for personalized advice based on your specific circumstances.

Category 4:

Procedure Information, Steps, & Techniques

(Includes Implant-Specific Knowledge)

28.06s

What's the difference between textured and smooth implants? Which is better?

Textured and smooth implants refer to the outer shell surface of the breast implants. Each type has its own characteristics, and the choice between them depends on several factors, including your anatomy, surgeon's recommendation, and personal preferences. Here's a comparison between textured and smooth implants:

Textured Implants:

- Textured implants have a rough surface created by texturing the outer shell of the implant.
- The texturing is designed to reduce the risk of capsular contracture (scar tissue formation) and minimize implant movement within the breast pocket.
- Some studies suggest that textured implants may have a lower risk of capsular contracture compared to smooth implants.
- Textured implants may be preferred in specific cases, such as breast reconstruction or when the implant is placed in a subglandular pocket (above the chest muscle).

Smooth Implants:

- Smooth implants have a smooth outer shell surface.
- They tend to have a softer feel and a more natural movement within the breast.
- Smooth implants may have a slightly lower risk of visible rippling or wrinkling compared to textured implants.
- They are commonly used in breast augmentation procedures, especially when the implant is placed in a submuscular pocket (below the chest muscle).

It's important to note that the choice between textured and smooth implants should be based on your individual circumstances and in consultation with your plastic surgeon. They will assess factors such as your desired outcome, anatomical considerations, and the potential risks and benefits associated with each type of implant.

The concept of "better" is subjective and depends on various factors. Your plastic surgeon will evaluate your unique situation, discuss the advantages and disadvantages of each implant type, and make recommendations based on their expertise and your goals. It's essential to have an open and thorough discussion with your surgeon to ensure the most appropriate choice for your specific case.

Category 4:

31.52s

Procedure Information, Steps, & Techniques

(Includes Implant-Specific Knowledge)

Will I keep the same implants forever, or will I need my implants changed in the future?

Breast implants are not considered lifetime devices, and there is a possibility that they may need to be replaced or removed at some point in the future. The duration of breast implant lifespan can vary among individuals, and factors such as implant type, age, lifestyle, and changes in your body over time can influence the need for revision or replacement. It's important to be aware of the following considerations:

1. Longevity: Breast implants are designed to be durable, but they are not expected to last a lifetime. The exact lifespan of implants is difficult to predict, as it can vary from person to person. On average, some studies suggest that breast implants may last 10 to 20 years or longer, but others have reported higher rates of revision or removal within that timeframe.
2. Implant-related Issues: Over time, breast implants may be subject to wear and tear, and potential issues can arise. These can include implant rupture or leakage, changes in implant position or shape, development of capsular contracture, or aesthetic concerns.
3. Aging and Body Changes: As you age, your body and breasts may change naturally. These changes, such as weight fluctuations, pregnancy, breastfeeding, and natural aging processes, can impact the appearance and position of the breasts, potentially leading to a desire for revision or removal of implants.

It's important to maintain regular follow-up appointments with your plastic surgeon for monitoring and assessment of your breast implants. Your surgeon will evaluate the condition of your implants, address any concerns you may have, and provide guidance on when or if revision surgery or implant replacement may be necessary.

It's worth noting that not all individuals will require revision or replacement of their breast implants. Some people may choose to have their implants removed or replaced for personal reasons, such as changes in preferences or lifestyle. Ultimately, the decision to undergo revision or replacement surgery will depend on your specific circumstances and goals, and it should be made in consultation with your plastic surgeon.

Category 5:

Patient Assessment

23.61s

If I upload pictures of myself, can you recommend to me which type of implants, size, and specific type of breast augmentation technique that would be best for me?

As an AI language model, I'm not able to directly view or analyze images or provide personalized recommendations based on specific pictures. Additionally, making recommendations for breast implants and surgical techniques requires a comprehensive evaluation of your individual anatomy, goals, and a detailed consultation with a qualified plastic surgeon.

To determine the best type of implants, size, and breast augmentation technique for you, it's important to consult with a board-certified plastic surgeon who can assess your unique circumstances. During the consultation, the surgeon will evaluate factors such as your current breast size and shape, chest wall anatomy, skin quality, and discuss your desired outcome.

During the consultation, your surgeon will consider your goals, take measurements, and conduct a physical examination. They may also use 3D imaging technology or other tools to simulate different implant sizes and projections, which can help you visualize potential outcomes.

Through this comprehensive evaluation, the surgeon will provide personalized recommendations based on their expertise and experience. They will take into account factors such as your desired breast size, body proportions, tissue characteristics, and your overall health.

It's important to have an open and detailed discussion with your plastic surgeon to ensure that your expectations align with realistic outcomes and to address any questions or concerns you may have. They will guide you in making informed decisions about the best approach for your breast augmentation procedure.

Remember that choosing the right surgeon is essential for achieving the best results. Make sure to select a board-certified plastic surgeon with experience in breast augmentation and a strong reputation for delivering safe and satisfactory outcomes.

Category 6:

Preparation for Surgery

36.82s

As a 27-year-old-female who drinks occasionally, and vapes daily, with ADHD (on medications), and well-controlled Crohn's disease (on medications), is there anything I can do to prepare for this surgery and make myself the best candidate?

To prepare for breast augmentation surgery and optimize your candidacy, here are some general guidelines to consider:

1. Consult with your Healthcare Providers: Before undergoing any surgery, it's essential to inform your plastic surgeon about your medical history, including your ADHD, medications, and well-controlled Crohn's disease. They will work closely with your healthcare providers to ensure your safety and make any necessary adjustments to your medications or surgical plan.
2. Follow a Healthy Lifestyle: Leading up to your surgery, it's important to maintain a healthy lifestyle. This includes eating a balanced diet, engaging in regular exercise (if approved by your healthcare providers), getting sufficient sleep, and avoiding smoking and recreational drug use. These practices can help support your overall well-being and facilitate a smoother recovery process.
3. Communicate Your Expectations: Clearly communicate your goals and expectations to your plastic surgeon during the consultation. This will help ensure that your surgeon understands your desired outcome and can provide appropriate recommendations tailored to your preferences.
4. Manage Medications: Follow your surgeon's instructions regarding the management of your ADHD medications and any other prescribed medications. It may be necessary to adjust the timing or dosage of certain medications to minimize potential risks during and after surgery. Your surgeon will coordinate with your healthcare providers to develop an appropriate plan.
5. Quit Smoking and Vaping: Smoking and vaping can have negative effects on the healing process and increase the risk of complications. If you currently smoke or vape, it is highly recommended to quit at least a few weeks before surgery and throughout the recovery period. Your surgeon can provide resources and support to help you quit if needed.
6. Arrange Support: It's beneficial to have a support system in place during your recovery period. Arrange for someone to assist you with daily activities, such as transportation, household chores, and personal care, especially during the initial days after surgery.
7. Follow Pre- and Post-operative Instructions: Your plastic surgeon will provide you with detailed pre-operative instructions, which may include guidelines on fasting before surgery, avoiding certain medications, and skin preparation. After surgery, carefully follow your surgeon's post-operative instructions regarding medication, dressings, activities, and follow-up appointments.

Remember, these guidelines are general in nature, and it's important to consult with your plastic surgeon for personalized advice based on your specific medical history and needs. They will guide you through the pre-operative preparations and ensure your safety and optimal outcomes.

Category 6:

Preparation for Surgery

38.88s

If I decide to get a breast augmentation, what can I expect on the lead up to my surgery?

If you decide to undergo a breast augmentation surgery, here's what you can generally expect during the lead-up to your surgery:

1. Initial Consultation: You will schedule an initial consultation with a board-certified plastic surgeon. During this consultation, you will discuss your goals, medical history, and any concerns or questions you may have. The surgeon will evaluate your breast anatomy, take measurements, and recommend appropriate options based on your desired outcome and unique characteristics.
2. Pre-operative Assessment: If you decide to proceed with the surgery, you will undergo a pre-operative assessment. This may include a physical examination, medical tests (such as blood tests or imaging), and a review of your overall health. The purpose of the assessment is to ensure that you are in good health and to identify any potential risk factors that need to be addressed before the surgery.
3. Planning the Procedure: Your plastic surgeon will discuss the details of the procedure, including the choice of implant type, size, incision placement, and implant placement (subglandular, submuscular, etc.). They will explain the benefits and potential risks associated with each option and help you make informed decisions.
4. Pre-operative Instructions: Your surgeon will provide you with specific pre-operative instructions to follow in the days leading up to your surgery. This may include guidelines on fasting before surgery, avoiding certain medications (such as blood thinners), and instructions for skin preparation. It's important to follow these instructions carefully to ensure your safety and optimize the surgical outcome.
5. Anesthesia Consultation: If general anesthesia will be used during the surgery, you may have a separate consultation with an anesthesiologist. They will review your medical history, discuss the anesthesia plan, and address any concerns related to anesthesia.
6. Financial and Scheduling Arrangements: You will work with the surgical facility to finalize financial arrangements, such as the cost of the procedure, payment options, and insurance coverage (if applicable). You will also schedule the surgery date and arrange for any necessary time off from work or other commitments during the recovery period.
7. Preparing for Recovery: Your surgeon will provide you with detailed instructions on how to prepare for your recovery period. This may include arranging for a responsible adult to accompany you to and from the surgical facility, preparing a comfortable recovery area at home, and gathering necessary supplies (such as post-operative garments, pain medications, and wound care materials).

It's important to note that the specific details of your journey may vary depending on your individual circumstances and the recommendations of your plastic surgeon. Following your surgeon's instructions and maintaining open communication with your healthcare team will help ensure a smooth and successful surgical experience.

56.62s

Category 7:

Recovery and Post-Procedural Instructions

What is recovery like to be able to do my normal day-to-day activities? How long will I need assistance for? How long before I can have intercourse? How long before I can go out in public without people knowing I had surgery? How long before I can return to work, and before I can start working out?

Recovery from breast augmentation surgery can vary from person to person, and the timeline for returning to normal day-to-day activities can differ. Here are some general guidelines, but it's important to note that your surgeon's specific instructions should always be followed:

1. Recovery Timeline: The initial recovery period after breast augmentation surgery typically lasts a few weeks. However, full recovery and the ability to resume all normal activities may take several months. Here's a general timeline:
   - First few days: You may experience soreness, swelling, and discomfort. Pain medication prescribed by your surgeon can help manage any discomfort.
   - First week: Rest and limit physical activity. You may need assistance with daily tasks during this time.
   - 1 to 2 weeks: Most individuals can resume light activities and return to work, depending on the nature of your job. Avoid heavy lifting or strenuous exercise during this period.
   - 4 to 6 weeks: Swelling and bruising should significantly subside, and most individuals can gradually increase activity levels. You may be able to engage in light workouts, but consult with your surgeon before starting any exercise routine.
   - 6 to 8 weeks: By this time, many individuals feel comfortable resuming most normal activities, including more intense exercise. However, always follow your surgeon's guidance regarding exercise and physical exertion.
2. Assistance and Intercourse: The level of assistance you need during the recovery period will depend on your individual circumstances. In the first few days after surgery, you will likely require someone's help with daily activities. As your recovery progresses, you may gradually regain your independence. Discuss your specific needs with your surgeon and plan for assistance accordingly.
   Regarding intercourse, it is typically recommended to wait until you have fully healed and received clearance from your surgeon. This is usually around 4 to 6 weeks after surgery, but your surgeon will provide specific guidelines based on your progress and healing.
3. Public Appearance: Swelling and bruising can be present immediately after surgery and gradually diminish over time. Within the first few weeks, most of the swelling should subside, and you can start going out in public without people readily noticing that you had surgery. However, individual healing rates can vary, so it's important to be patient and follow your surgeon's advice on when it is appropriate to resume social activities.
4. Return to Work and Exercise: The timing for returning to work depends on the physical demands of your job and how you are healing. In jobs that are not physically demanding, many individuals can return to work within 1 to 2 weeks after surgery. If your job involves strenuous activity or heavy lifting, you may need to wait longer before resuming work duties.
   The timeline for returning to exercise and working out will depend on your individual progress and your surgeon's recommendations. Light exercise, such as walking, can typically be resumed after a few weeks, while more intense workouts may need to be postponed for 4 to 6 weeks or longer. It's crucial to consult with your surgeon to receive personalized guidance on when it is safe to resume exercise and physical activities.

Keep in mind that these timelines are general estimates, and individual recovery experiences may vary. Your surgeon will provide you with specific instructions tailored to your unique case and closely monitor your progress during follow-up appointments. It's important to follow their guidance and allow your body ample time to heal for the best results.

Category 7:

Recovery and Post-Procedural Instructions

Will I still need to wear bras after a breast augmentation either right after the surgery or long term?

After breast augmentation surgery, your surgeon will provide specific instructions regarding the use of bras during the recovery period and beyond. Here are some general guidelines:

1. Immediately After Surgery: Right after the surgery, your surgeon will likely provide you with a surgical bra or compression garment to wear. This garment helps support the breasts, minimize swelling, and promote healing. You will typically wear this special bra for a few weeks until instructed otherwise by your surgeon.
2. Post-Recovery Period: Once you have healed sufficiently, your surgeon may recommend transitioning to a supportive, non-underwire bra. The type and style of bra will depend on your individual needs and preferences. Your surgeon may advise you to wear a supportive bra during the day and at night for several months to help maintain the shape and position of your implants.
3. Long-Term Bra Use: Whether you will need to wear bras long-term after breast augmentation surgery can vary. Some individuals with breast implants continue to wear bras for additional support and comfort, while others may feel comfortable going without a bra at times. This will depend on factors such as breast tissue characteristics, implant size, and personal comfort.

It's important to discuss bra usage with your surgeon during your consultation and follow-up appointments. They will provide guidance based on your specific circumstances, including recommendations on the type of bra, level of support needed, and any specific instructions for long-term bra use.

Remember that wearing well-fitted and supportive bras can help maintain the aesthetic results of your breast augmentation and provide added comfort. It's also important to choose bras that do not put excessive pressure on the incision sites and avoid underwire bras until your surgeon gives the approval for their use.

Additionally, it's worth noting that breast shape and position can change over time due to factors such as aging, weight fluctuations, and natural breast tissue changes. Regular follow-up appointments with your surgeon will help monitor your results and address any concerns or adjustments that may be necessary.

38.03s

Category 8:

Procedure Costs & Surgeon Recommendations

How much does a breast augmentation cost? Can it be covered by insurance? Can you recommend to me the best surgeon I can go to near my area?

The cost of breast augmentation surgery can vary depending on several factors, including the surgeon's experience, geographic location, type of implants used, surgical facility fees, anesthesia fees, and additional expenses such as pre-operative tests and post-operative garments. On average, the cost of breast augmentation in the United States can range from $5,000 to $10,000 or more.

Insurance coverage for breast augmentation is generally limited to cases where it is deemed medically necessary. This may include breast reconstruction after mastectomy or breast asymmetry correction. However, cosmetic breast augmentation for purely aesthetic reasons is typically not covered by insurance.

To find a reputable plastic surgeon near your area, consider the following steps:

1. Research: Use online resources such as the American Society of Plastic Surgeons (ASPS) or the American Society for Aesthetic Plastic Surgery (ASAPS) to find board-certified plastic surgeons in your area. These organizations have directories that can help you identify qualified surgeons.
2. Read Reviews: Look for patient reviews and testimonials to gain insight into the experiences of others who have undergone breast augmentation with specific surgeons. This can provide an idea of patient satisfaction and overall quality of care.
3. Consultations: Schedule consultations with multiple surgeons to discuss your goals, ask questions, and evaluate their expertise and approach. During the consultation, pay attention to the surgeon's communication style, willingness to address your concerns, and their ability to explain the procedure in a clear and comprehensive manner.
4. Credentials and Experience: Verify that the surgeon is board-certified by the American Board of Plastic Surgery (ABPS) or an equivalent organization in your country. Board certification indicates that the surgeon has met rigorous standards of training, experience, and ethical practice. Additionally, consider the surgeon's experience in performing breast augmentation procedures and their specialization in aesthetic breast surgery.
5. Before-and-After Photos: Ask to see before-and-after photos of the surgeon's previous breast augmentation patients. This will give you an idea of their skill, technique, and the quality of their results. Pay attention to patients with similar starting conditions or desired outcomes to yours.
6. Personal Connection: Choose a surgeon with whom you feel comfortable and confident. It's important to establish a good rapport and have open communication with your surgeon throughout the process.

Remember to prioritize the expertise, experience, and reputation of the surgeon over cost when making your decision. Cosmetic surgery is an investment in your well-being and aesthetic goals, and selecting a qualified and reputable surgeon is crucial for achieving safe and satisfactory outcomes.
