# Supplementary material for: Preoperative Patient Guidance and Education in Aesthetic Breast Plastic Surgery: A Novel Proposed Application of Artificial Intelligence Large Language Models
Source: Aesthet Surg J Open Forum. 2024 Aug 13;6:ojae062. doi: 10.1093/asjof/ojae062 (PMC11385898; doi:10.1093/asjof/ojae062)
Supplement: ojae062_Supplementary_Data [file ojae062_supplementary_data.zip › Appendix B.pdf]

23.78s

Category 1:

Aesthetic Needs Inquiry; Awareness of Available Procedures

I have some questions about breast plastic surgery I'd like to ask you. Feel free to ask me more questions if you need, so you can give me the best answers. I'm interested in lifting my breast and restoring their appearance after having kids. I bothered by how deflated and saggy my breasts look. What are my options?

I'd be happy to provide information about breast plastic surgery options for lifting and restoring the appearance of your breasts after having kids. Before proceeding, it's important to note that the best course of action would be to consult with a board-certified plastic surgeon who can assess your specific situation and provide personalized recommendations. Nonetheless, here are some common options that are often considered:

1. Breast Lift (Mastopexy): This procedure is designed to raise and reshape sagging breasts by removing excess skin and tightening the surrounding tissue. It can help restore a more youthful and lifted appearance to the breasts. A breast lift alone does not significantly change the size of the breasts, but it can be combined with other procedures if desired.
2. Breast Augmentation: If you're also concerned about the volume loss in your breasts, you may consider breast augmentation. This procedure involves placing implants to enhance the size and shape of the breasts. Implants can provide additional fullness and improve the overall contour of the breasts. Breast augmentation can be performed in conjunction with a breast lift to address both volume and sagging concerns.
3. Combination of Breast Lift and Augmentation: This option is commonly chosen when women desire both lifting and restoring volume to their breasts. By combining a breast lift with augmentation, you can achieve a more comprehensive improvement in shape, size, and position.

During a consultation with a plastic surgeon, they will assess your individual needs, examine the current state of your breasts, and discuss your aesthetic goals. They will then recommend the most appropriate procedure or combination of procedures to help you achieve your desired outcome. It's important to have a clear understanding of the potential risks, recovery process, and expected results before making a decision.

Remember, every individual is unique, and the suitability of these procedures may vary depending on factors such as your overall health, breast anatomy, and personal preferences. Consulting with a qualified plastic surgeon will provide you with the most accurate and tailored advice for your specific situation.

Category 2:

Patient Candidacy and Procedure Indications

I'm a 46-year-old-female. I have rheumatoid arthritis, but it's well controlled on medications. I have some anxiety. I don't drink or smoke cigarettes, but I do smoke cannabis every day to relax me before bed. Am I a good candidate for a mastopexy?

As an AI language model, I cannot provide a definitive answer regarding your candidacy for a mastopexy (breast lift) without a thorough evaluation by a qualified plastic surgeon. However, I can offer some general considerations based on the information you provided.

Your age alone does not exclude you from being a candidate for a mastopexy. Many women in their 40s and beyond undergo this procedure successfully. However, the overall health and specific medical conditions you have may influence the decision. In your case, having well-controlled rheumatoid arthritis on medications is a positive factor. However, it's important to disclose your medical history and medications to your plastic surgeon during the consultation, as certain medications or medical conditions may have implications for the surgical process and recovery.

Regarding your anxiety, it's crucial to discuss this with your plastic surgeon as well. Anxiety can impact your overall well-being and may influence your experience during the surgery and recovery period. Your surgeon can provide guidance on how to manage anxiety and ensure a smooth process.

Regarding your cannabis use, it's essential to be transparent with your surgeon about any substances you consume, including cannabis. Although smoking cannabis may not necessarily disqualify you from undergoing a mastopexy, it's important to be aware of potential interactions between cannabis and anesthesia or medications used during and after surgery. Your surgeon will provide specific instructions on when to discontinue cannabis use before and after the procedure to minimize any risks.

Ultimately, the decision about your candidacy for a mastopexy will be made by a qualified plastic surgeon who can consider your medical history, current health, individual circumstances, and goals. It is crucial to have an open and honest discussion with your surgeon, as they will have the expertise to assess your suitability for the procedure and address any concerns you may have.

Category 3:

Procedure Safety and Risks

30.70s

What are the risks associated with mastopexy? Can you give me specific estimates, and an overall complication rate?

Mastopexy, like any surgical procedure, carries certain risks and potential complications. It's important to have a thorough discussion with a board-certified plastic surgeon to fully understand the risks involved. While I can provide you with a general overview of potential complications, specific estimates and overall complication rates can vary widely depending on individual factors and surgical techniques used. It's best to consult with a plastic surgeon who can provide you with more accurate information based on your unique situation.

Some potential risks and complications associated with mastopexy may include:

1. Scarring: Mastopexy involves incisions, resulting in scars. The extent of scarring can vary depending on the technique used and individual healing characteristics. Scars may fade over time but will not completely disappear.
2. Changes in nipple sensation: Temporary or permanent changes in nipple sensation can occur after a breast lift. Numbness, increased sensitivity, or decreased sensation in the nipple or surrounding areas may be experienced.
3. Infection: Infections can occur after any surgery. Steps are taken to minimize this risk, including the use of sterile techniques and antibiotics. However, infections may still occur and may require treatment with antibiotics or, in rare cases, additional surgery.
4. Hematoma or seroma: Collection of blood (hematoma) or fluid (seroma) can occur after surgery. These may require drainage or additional procedures to resolve.
5. Changes in breast or nipple shape: While mastopexy aims to improve breast shape and position, there is a possibility of asymmetry, changes in shape, or irregularities. Additional revisions may be needed to achieve desired results.
6. Loss of nipple or breast tissue: In rare cases, blood flow to the nipple or breast tissue may be compromised, leading to tissue necrosis (death of tissue). This risk is higher in smokers or individuals with certain medical conditions.
7. Anesthesia risks: General anesthesia carries its own set of risks, such as reactions to medications or complications related to the administration of anesthesia.

These are just a few examples, and there may be other potential risks and complications associated with mastopexy. It's essential to discuss these in detail with your plastic surgeon and carefully follow their pre- and post-operative instructions to minimize risks and enhance your recovery.

Remember that the expertise and experience of the surgeon performing the procedure can also impact the likelihood of complications. Choosing a qualified and board-certified plastic surgeon will help reduce the risks associated with mastopexy.

Category 3:

Procedure Safety and Risks

22.89s

Can I breastfeed after a mastopexy if I get pregnant again?

The ability to breastfeed after a mastopexy (breast lift) can vary from person to person. While some women may still be able to breastfeed after the procedure, others may experience a decrease in milk production or changes in nipple sensation that can affect breastfeeding.

During a breast lift, the nipple and areola are repositioned to achieve a more youthful appearance. This repositioning may involve the detachment and reattachment of milk ducts, which can potentially impact milk flow. Additionally, the surgery may alter the sensitivity of the nipples, which can also affect the breastfeeding experience.

It's important to discuss your desire to breastfeed in the future with your plastic surgeon during the consultation. They can provide personalized advice based on your individual circumstances and the specific surgical techniques planned.

While it's not possible to predict with certainty how a mastopexy will impact your ability to breastfeed, it's worth noting that some women who have undergone breast lifts have successfully breastfed their children. However, there is also a chance that you may experience challenges or a decrease in milk supply. If breastfeeding is an important consideration for you, it's best to discuss this thoroughly with your surgeon and explore all available options.

In general, it's recommended to wait until you have completed your family planning and finished having children before undergoing a mastopexy. This allows you to avoid potential complications and maximize the longevity of your surgical results. However, every individual's circumstances are unique, so discussing your specific goals and concerns with a qualified plastic surgeon will provide you with the most accurate information and guidance regarding breastfeeding after a mastopexy.

Category 3:

30.24s

Procedure Safety and Risks

Will mastopexy affect my chances of getting breast cancer, or affect my ability to get mammograms and detect breast cancer in the future? Do I still need to get mammograms?

Undergoing a mastopexy (breast lift) does not directly affect your chances of developing breast cancer. However, it's important to note that breast cancer risk is influenced by various factors, including age, family history, genetic predisposition, hormonal factors, and lifestyle choices. It's advisable to discuss your personal risk factors and any concerns you may have with your healthcare provider or a breast specialist.

Regarding mammograms, the ability to detect breast cancer through mammography can be influenced by breast surgeries, including mastopexy. The rearrangement of breast tissue and scarring from the procedure can potentially make it more challenging to interpret mammogram results accurately. However, this does not mean that you should forego mammograms.

It is still important to continue routine breast cancer screening and follow the recommended guidelines for mammography based on your age and risk factors. When you schedule a mammogram, it's crucial to inform the healthcare professionals about any breast surgeries you have undergone, including a mastopexy. This information will help the radiologist and technicians interpret the images appropriately and take necessary precautions.

In some cases, additional imaging techniques, such as ultrasound or MRI, may be recommended to complement mammography and provide a more comprehensive evaluation of breast tissue. Your healthcare provider will determine the most appropriate screening approach based on your individual situation.

Regular self-breast exams and clinical breast exams by a healthcare professional are also important components of breast health monitoring. It's essential to remain vigilant and promptly report any changes or concerns to your healthcare provider.

Overall, while mastopexy can impact mammogram interpretation, it does not negate the need for breast cancer screening. It's crucial to maintain regular breast health examinations and follow the recommended guidelines for mammography to ensure early detection and effective management of breast cancer, if necessary.

Category 3:

19.15s

Procedure Safety and Risks

Is it safe to combine a mastopexy with other plastic surgery procedures I may need?

Combining a mastopexy (breast lift) with other plastic surgery procedures is a common practice and can be safe when performed by a skilled and experienced plastic surgeon. Many women choose to combine a mastopexy with procedures such as breast augmentation, liposuction, or abdominoplasty (tummy tuck) to achieve comprehensive aesthetic improvements.

However, it's important to note that combining multiple procedures does increase the complexity and duration of the surgery, which can potentially increase the risk of complications. Your plastic surgeon will carefully assess your health, medical history, and individual circumstances to determine if you are a suitable candidate for combined procedures.

During the consultation, you should openly discuss your goals and desires with your plastic surgeon. They will evaluate your specific situation, consider factors such as your overall health, the extent of the procedures, and the potential risks and benefits associated with the combination of surgeries. They will also take into account factors such as the anticipated length of surgery, anesthesia considerations, and the expected recovery process.

Your surgeon will provide personalized recommendations and help you understand the potential risks and benefits of combining procedures. They will also discuss the expected recovery timeline, potential limitations, and any additional precautions that may need to be taken.

Remember that the decision to combine procedures should be based on your individual circumstances and the expertise and guidance of your plastic surgeon. They will have the best knowledge of your unique situation and can provide professional advice on the safety and appropriateness of combining a mastopexy with other plastic surgery procedures.

Category 4:

Procedure Information, Steps, & Techniques

31.30s

Can you explain to me the steps of how a mastopexy is performed?

Certainly! Here is a general overview of the steps involved in a mastopexy (breast lift) procedure:

1. Anesthesia: The first step is administering anesthesia. You will typically be given general anesthesia, which means you will be asleep and pain-free throughout the surgery. Alternatively, in some cases, local anesthesia with sedation may be used.
2. Incision Placement: The plastic surgeon will then create incisions based on the agreed-upon surgical technique and your specific needs. The incision pattern will depend on the degree of breast sagging, desired outcome, and other factors. Common incision patterns include:
   - Around the areola (periareolar): This incision is made around the outer edge of the areola.
   - Around the areola and vertically down to the breast crease (lollipop or keyhole incision): This incision combines a periareolar incision with a vertical incision.
   - Around the areola, vertically down to the breast crease, and horizontally along the breast crease (anchor incision): This incision pattern is often used for more extensive corrections.
3. Reshaping and Lifting: After making the incisions, the surgeon will lift and reshape the breast tissue to achieve the desired contour and position. Excess skin is removed, and the remaining skin is tightened to create a firmer appearance. If desired, the surgeon may also reposition the nipple and areola to a higher position on the breast mound.
4. Areola Reduction (if necessary): In some cases, the size of the areola may be reduced to match the new breast shape and size. This involves removing a small amount of the outer edge of the areola.
5. Closing Incisions: Once the desired reshaping and lifting are achieved, the surgeon will close the incisions using sutures. The incisions are usually layered, with deep sutures in the breast tissue and skin sutures or surgical adhesive to close the skin.
6. Dressing and Supportive Garments: After the incisions are closed, the surgeon will apply dressings to protect the incision sites and may place a supportive bra or surgical garment to provide additional support and help with healing.

The specific techniques used may vary depending on the surgeon's approach and your unique needs. It's important to discuss the details of the surgical plan with your plastic surgeon during the consultation to have a clear understanding of the steps involved in your specific mastopexy procedure.

Remember, this is a general overview, and the actual procedure may differ based on individual factors and the surgeon's preferences.

28.20s

Category 4:

Procedure Information, Steps, & Techniques

Where will my scars be? Do some of the techniques you mentioned give more scars than others? I would like the least amount of scars possible and the best lift.

The placement of scars in a mastopexy procedure will depend on the surgical technique chosen, the degree of breast sagging, and the specific needs of the patient. It's important to note that some scarring is inevitable with any breast lift procedure, as incisions are necessary to remove excess skin and reshape the breasts.

Here are the common incision patterns and their corresponding scar locations:

1. Periareolar Incision: This incision is made around the outer edge of the areola. It results in a scar that encircles the areola. This technique is often suitable for patients with mild sagging or those who require minimal lifting.
2. Lollipop or Keyhole Incision: This incision combines a periareolar incision with a vertical incision that extends downward from the areola to the breast crease. The resulting scar forms a lollipop shape, with a circular scar around the areola and a vertical scar below it. This technique is typically used for moderate sagging and provides more extensive reshaping and lifting.
3. Anchor Incision: The anchor incision involves three components: a periareolar incision, a vertical incision from the areola to the breast crease, and a horizontal incision along the breast crease. The resulting scar resembles an anchor shape, with scars around the areola, vertically below it, and horizontally along the breast crease. This technique is often employed for significant breast sagging or when a more substantial lift is needed.

While each technique has its advantages, it's important to note that the amount of scarring is typically proportional to the degree of correction needed. More extensive sagging may require a larger incision pattern to achieve the desired lift and reshaping.

To minimize scarring, it's crucial to follow post-operative care instructions provided by your plastic surgeon. This may include proper wound care, avoiding excessive sun exposure on the incision sites, and using scar-minimizing techniques such as silicone gel or sheets as recommended by your surgeon.

During your consultation, your plastic surgeon will assess your breast anatomy, degree of sagging, and your desired outcome. They will recommend the most appropriate technique to achieve the desired lift while considering your scarring concerns. They will also explain the expected location and characteristics of the scars, providing you with a realistic expectation of the outcome.

Remember that while scarring is inevitable, most scars tend to fade and become less noticeable over time with proper care and healing.

Category 5:

Patient Assessment

16.11s

If I upload pictures of myself, can you recommend to me which type of mastopexy is best for me?

I'm sorry, but as an AI language model, I cannot process or analyze images or provide personalized medical advice. I can only offer general information and guidance. Determining the most suitable mastopexy technique for you requires a comprehensive evaluation by a qualified plastic surgeon who can assess your specific needs, anatomy, and desired outcome.

I recommend consulting with a board-certified plastic surgeon for a thorough examination and discussion of your goals. They will evaluate factors such as the degree of breast sagging, breast volume, nipple position, and overall breast anatomy to determine the most appropriate mastopexy technique for your specific situation.

During your consultation, your plastic surgeon will assess your individual needs and discuss the various options available to achieve your desired lift and shape. They will explain the pros and cons of each technique, taking into account factors such as scarring, the extent of lifting required, and the expected outcomes.

It's important to have an open and honest conversation with your plastic surgeon, sharing your goals, concerns, and any questions you may have. They will provide you with personalized recommendations and guide you through the decision-making process, ensuring that you have a clear understanding of the best approach for your mastopexy.

Remember, the expertise and experience of a qualified plastic surgeon are essential in determining the most appropriate technique for your specific needs.

Category 6:

Preparation for Surgery

32.54s

As a 46-year-old-female with rheumatoid arthritis, anxiety, who doesn't drink or smoke cigarettes, but smokes cannabis daily is there anything I can do to prepare for this surgery?

If you are considering mastopexy surgery and have certain medical conditions and habits, it's important to take steps to prepare yourself and optimize your chances of a successful outcome. Here are some general recommendations to consider:

1. Consultation with Your Plastic Surgeon: Schedule a consultation with a board-certified plastic surgeon who has experience with mastopexy and discuss your medical history, including your rheumatoid arthritis, anxiety, and cannabis use. They will evaluate your overall health and provide personalized recommendations.
2. Medication Management: Inform your plastic surgeon about all medications you are currently taking, including those for rheumatoid arthritis and anxiety. They will assess the potential impact of these medications on the surgery and provide guidance on how to manage them before, during, and after the procedure.
3. Rheumatoid Arthritis Management: Ensure that your rheumatoid arthritis is well controlled with medications and follow the advice of your rheumatologist. It's important to discuss any concerns about potential interactions between rheumatoid arthritis medications and anesthesia or post-operative healing with both your rheumatologist and plastic surgeon.
4. Smoking and Cannabis Use: While you mentioned that you don't smoke cigarettes, it's worth noting that smoking, in any form, can have negative effects on the healing process. It's recommended to refrain from smoking cannabis or using any nicotine products for a certain period before and after the surgery as advised by your plastic surgeon. Smoking can impair wound healing and increase the risk of complications. Discuss your cannabis use openly with your surgeon, as they may have specific guidelines or recommendations based on your situation.

6. Emotional Well-being: Given your mention of anxiety, it's important to prioritize your emotional well-being. Engage in stress-management techniques, such as meditation, relaxation exercises, or therapy, to help manage anxiety before and after the surgery. Communicate your concerns and anxieties with your plastic surgeon and their team, as they can provide support and guidance.
7. Follow Pre-Operative Instructions: Your plastic surgeon will provide you with specific pre-operative instructions to follow before your mastopexy surgery. These may include guidelines for fasting, avoiding certain medications or supplements, and ensuring you have any necessary post-operative care items ready. It's crucial to adhere to these instructions to minimize potential risks and complications.

Remember, these are general recommendations, and it's important to consult with your plastic surgeon directly for personalized advice and guidelines based on your unique situation. They will guide you through the pre-operative preparation process and provide you with the specific instructions you need to follow for a successful surgery and recovery.

Category 6:

Preparation for Surgery

24.96s

If I decide to get a mastopexy, what can I expect in the lead up to my surgery?

If you decide to proceed with a mastopexy (breast lift) surgery, here's an overview of what you can generally expect in the lead up to your surgery:

1. Initial Consultation: You will have an initial consultation with a board-certified plastic surgeon. During this consultation, you will discuss your goals, medical history, current medications, and any concerns you may have. The surgeon will examine your breasts, assess your suitability for the procedure, and provide recommendations tailored to your specific needs.
2. Pre-operative Evaluation: Before the surgery, you will typically undergo a pre-operative evaluation, which may include blood tests, a physical examination, and possibly additional imaging such as mammography or breast ultrasound. These evaluations help ensure you are in good health and identify any potential risks or factors that need to be taken into consideration during the surgery.
3. Discussion of Surgical Plan: Your plastic surgeon will explain the surgical plan in detail, including the chosen technique, incision pattern, expected outcomes, and potential risks and complications. They will also discuss the expected recovery process and answer any questions you may have. Make sure to have a clear understanding of the procedure and communicate your expectations and concerns.
4. Pre-operative Instructions: Your surgeon will provide you with specific pre-operative instructions to follow before your surgery. These instructions may include guidelines on fasting before surgery, discontinuing certain medications or supplements that can increase bleeding risks, and showering with a specialized antibacterial soap prior to the procedure. It's crucial to adhere to these instructions to ensure your safety and optimize the surgical outcome.
5. Consent and Financial Arrangements: Before the surgery, you will be asked to sign a consent form, acknowledging your understanding of the procedure, potential risks, and expected outcomes. You will also discuss financial arrangements, including the cost of the surgery, payment options, and any necessary insurance coverage.
6. Preparing for Recovery: In the lead up to the surgery, you may need to make some preparations for your recovery. This may include arranging for someone to drive you home after the surgery and arranging for assistance with daily activities during the initial recovery period. Your surgeon will provide you with specific instructions on what to expect during the recovery phase and how to prepare accordingly.

It's important to note that the specifics of your pre-operative journey may vary based on your individual circumstances and the protocols of your plastic surgeon. Make sure to maintain open communication with your surgeon, follow their instructions, and address any concerns you may have leading up to your mastopexy surgery.

31.87s

Category 7:

Recovery and Post-Procedural Instructions

What is recovery like to be able to do my normal day-to-day activities? How long will I need assistance at home for? How long before I can have intercourse? How long before I can go out in public without people knowing I had surgery? How long before I can return to work, and before I can start working out?

The recovery process after a mastopexy (breast lift) can vary from person to person, but here are some general guidelines for the aspects you mentioned:

1. Normal Day-to-Day Activities: Initially, you will experience some discomfort, swelling, and restricted movement. You may need assistance with activities such as cooking, cleaning, and lifting for the first few days or even up to a week, depending on the extent of the surgery and your individual recovery. It is essential to follow your surgeon's post-operative instructions regarding activity restrictions and gradually increase your level of physical activity as advised.
2. Assistance at Home: The duration of assistance at home will depend on your specific situation, the extent of the surgery, and your rate of recovery. Typically, it is advisable to have someone available to assist you for the first few days or up to a week, especially with tasks that require lifting or strenuous movements. This will ensure a smoother recovery and help you adhere to activity restrictions during the initial healing phase.
3. Intercourse: Your plastic surgeon will provide guidance on when it is safe to resume sexual activity after the surgery. It is generally recommended to wait until the initial healing has taken place and any discomfort or swelling has subsided. This period can vary, but typically it is advised to wait at least 4-6 weeks before engaging in sexual intercourse. However, it is crucial to consult with your surgeon for personalized advice based on your specific situation.
4. Going out in Public: The time it takes before you feel comfortable going out in public without others noticing that you had surgery can vary. Swelling and bruising typically peak in the first week or two and gradually subside over the following weeks. You may consider wearing loose-fitting, comfortable clothing to help conceal any bandages or surgical garments during the initial healing period. By around 2-4 weeks, many patients feel more confident in their appearance and are comfortable going out in public.
5. Return to Work: The time needed before returning to work depends on several factors, including the nature of your job, the extent of the surgery, and your individual recovery progress. In general, you may need to take around 1-2 weeks off work, especially if your job involves physical exertion or lifting. If your job is relatively sedentary, you may be able to return to work sooner, but it's important to consult with your surgeon for personalized recommendations based on your specific circumstances.
6. Exercise and Physical Activity: It's essential to allow your body enough time to heal before resuming any strenuous physical activity or exercise routines. Your plastic surgeon will provide specific instructions on when it is safe to start exercising again. In general, you may need to wait around 4-6 weeks before gradually incorporating light exercises. Strenuous activities and heavy lifting should be avoided for a longer period, typically up to 6-8 weeks or as advised by your surgeon.

Remember, these are general guidelines, and the actual recovery timeline can vary from person to person. Your plastic surgeon will provide you with personalized post-operative instructions, including guidelines for activity restrictions, wound care, and follow-up appointments. It's crucial to follow these instructions closely to ensure optimal healing and the best possible outcomes.

30.78s

Category 7:

Recovery and Post-Procedural Instructions

Will my breasts deflate or sag again after I get a mastopexy, or will they stay in the corrected position forever? What if I have kids again? What are all the things that can make my result and sagging worse over time so I know to avoid them, and is there anything I can do to best-maintain my results?

After a mastopexy (breast lift), your breasts will be lifted and reshaped to a more youthful position. However, it's important to understand that the aging process, lifestyle factors, and certain life events like pregnancy can still affect the appearance of your breasts over time. Here are some considerations:

1. Aging and Natural Changes: As you age, your skin will naturally lose some of its elasticity, and the effects of gravity may cause mild sagging to occur over time. While a mastopexy can provide long-lasting results, it cannot completely stop the aging process. However, your breasts will generally maintain a more youthful position compared to if you hadn't undergone the procedure.
2. Pregnancy and Breastfeeding: If you have children after a mastopexy, it can potentially impact the results. Pregnancy and breastfeeding can cause changes in breast volume and shape, and may result in some degree of breast sagging. It's advisable to discuss your plans for future pregnancies with your plastic surgeon during the consultation, as they can provide guidance on the timing of the procedure and discuss options to minimize potential changes in your breasts.
3. Weight Fluctuations: Significant weight gain or loss can affect the appearance of your breasts. Maintaining a stable and healthy weight is important to help preserve the results of your mastopexy. It's advisable to follow a balanced diet, engage in regular exercise, and maintain a healthy lifestyle overall.
4. Smoking and Sun Exposure: Smoking can negatively impact healing and increase the risk of complications. Additionally, excessive sun exposure without proper sun protection can lead to skin damage and affect the quality of your breast skin over time. It's best to quit smoking and protect your skin from excessive sun exposure to maintain the longevity of your mastopexy results.
5. Supportive Bras: Wearing supportive bras that fit well can help maintain the shape and support of your breasts. Your plastic surgeon may recommend wearing a specialized post-operative bra during the initial healing period. After that, choosing bras with proper support and fit can help minimize the effects of gravity and provide ongoing support to your lifted breasts.
6. Regular Check-ups: It's important to continue regular breast examinations and mammograms as recommended by your healthcare provider. This is important for breast health and to detect any potential changes or abnormalities that may occur over time.

While there are factors that can impact the longevity of your mastopexy results, following your plastic surgeon's post-operative instructions, maintaining a healthy lifestyle, and discussing any potential life events or concerns with your surgeon can help optimize and maintain your results as much as possible. It's important to have realistic expectations and understand that your breasts may undergo some natural changes over time, but a mastopexy can still provide long-lasting improvement in breast shape and position.

27.73s

Category 8:

Procedure Costs & Surgeon Recommendations

How much does a mastopexy cost? Can it be covered by insurance? Can you recommend to me the best surgeon I can go to near my area?

The cost of a mastopexy (breast lift) can vary depending on several factors, including the geographic location, the surgeon's experience and reputation, the complexity of the procedure, facility fees, anesthesia fees, and any additional procedures that may be performed in conjunction with the mastopexy. On average, the cost of a mastopexy in the United States can range from $5,000 to $15,000 or more.

Insurance coverage for a mastopexy is typically limited to cases where the procedure is deemed medically necessary rather than purely cosmetic. For example, if you have significant breast asymmetry or ptosis (sagging) that causes functional issues, insurance may cover a portion of the surgery. However, coverage criteria can vary between insurance plans, and it's best to consult with your insurance provider and your plastic surgeon to determine if your specific case qualifies for coverage.

When it comes to finding the best surgeon near your area, it's important to do thorough research and consider several factors. Here are some suggestions:

1. Board Certification: Look for a plastic surgeon who is board-certified by the American Board of Plastic Surgery or the relevant board in your country. Board certification indicates that the surgeon has met specific standards of training and expertise in plastic surgery.
2. Experience and Expertise: Seek a surgeon who specializes in breast surgeries and has extensive experience performing mastopexy procedures. Ask about their experience, view before and after photos of their previous patients, and inquire about their patient satisfaction rates.
3. Referrals and Reviews: Ask your primary care physician, friends, or family members for recommendations. Read online reviews and testimonials from patients who have had a mastopexy with the surgeon you are considering.
4. Consultations: Schedule consultations with multiple surgeons to discuss your goals, ask questions, and evaluate their approach and recommendations. Pay attention to their communication style, attentiveness to your concerns, and their ability to explain the procedure and potential outcomes.
5. Credentials and Hospital Affiliations: Check the surgeon's credentials, hospital affiliations, and any disciplinary actions or malpractice claims against them. Ensure that the surgeon operates in an accredited facility with a track record of safety and quality care.

Ultimately, the decision of choosing the best surgeon is a personal one. It's important to find a surgeon who not only has the necessary qualifications and experience but also makes you feel comfortable and confident in their abilities. Take your time, gather information, and choose a surgeon who aligns with your goals and values.
